# NOP Receptor Ligands as Potential Agents for Inflammatory and Autoimmune Diseases

**DOI:** 10.4061/2011/836569

**Published:** 2011-11-17

**Authors:** Elaine C. Gavioli, Pedro R. T. Romão

**Affiliations:** ^1^Laboratório de Farmacologia Comportamental, Programa de Pós-graduação em Desenvolvimento e Inovação Tecnológica em Medicamentos, Departamento de Biofísica e Farmacologia, Centro de Biociências, Universidade Federal do Rio Grande do Norte, 59072-970 Natal, RN, Brazil; ^2^Laboratório de Biologia Celular e Imunologia, Programa de Pós-Graduação em Ciências da Saúde, Departamento de Ciências Básicas da Saúde, Universidade Federal de Ciências da Saúde de Porto Alegre, 90050-170 Porto Alegre, RS, Brazil

## Abstract

Nociceptin/orphanin FQ (N/OFQ) is a seventeen-amino acid peptide that is the endogenous ligand of a G-protein-coupled receptor (NOP). Various immune cells express the precursor protein and secrete N/OFQ as well as display binding sites for this peptide. The functional capacity of NOP receptor was demonstrated *in vitro* and *in vivo* studies by the ability of N/OFQ to induce chemotaxis of immune cells, to regulate the expression of cytokines and other inflammatory mediators, and to control cellular and humoral immunity. In this context, N/OFQ could modulate the outcome of some inflammatory diseases, such as sepsis and autoimmune pathologies by mechanisms not clearly elucidated yet. In fact, human body fluid revealed increased levels of N/OFQ under sepsis, arthritis, and Parkinson's diagnose. Preclinical studies pointed to the blockade of NOP receptor signaling as successful in treating these experimental conditions. Further preclinical and clinical studies are required to investigate the potential of NOP ligands in treating inflammatory diseases.

## 1. N/OFQ and NOP Receptor

The peptide nociceptin/orphanin FQ (N/OFQ) was isolated for the first time in 1995 by two distinct research groups from rat [1] and porcine [2] brain extracts. N/OFQ is composed by seventeen amino acids residues, and this peptide was nominated nociceptin due to the hyperalgesic effects that it evokes after supraspinal administration [1]. The other name, orphanin FQ, was given by the Reinscheid's group due to the fact that this peptide displayed affinity to an orphan receptor—actually, this was one of the first well-experienced cases of reversal pharmacology; while F and Q were used to discriminate the amino acids phenylalanine from the N-terminal and glutamine located in the C-terminal position [2]. 

N/OFQ is a neuropeptide sharing sequence homology with classical opioid peptides, but with a distinct pharmacological profile. N/OFQ was being considered an opioid-like peptide, since it is structurally related to endogenous opioids, particularly dynorphin A; however, N/OFQ does not bind to classical opioid receptors [2]. Additionally, N/OFQ is the endogenous ligand for the N/OFQ peptide (NOP) receptor (nominated before as opioid receptor like-1, abbreviated as ORL-1), which is closely related to the opioid receptor family, but does not bind opioid ligands [3]. NOP, similar to the opioid family, is a receptor coupled to a G-protein. Thus, the activation of the N/OFQ signaling via NOP receptor can lead, among other effects, to inhibition of adenylate cyclase, blockade of Ca^2+^, and opening of K^+^ channels (for a review, see [4]), as described in details in Section 5.

The N/OFQ peptide precursor (ppN/OFQ) and NOP receptor are widely expressed in the nervous system as well as in peripheral organs and the immune system. In the central nervous system, of particular interest is the expression of NOP receptor in the forebrain, including cortical areas, olfactory regions, the thalamus, and a variety of limbic structures, such as the hippocampus, the amygdaloid complex, and in several nuclei of the hypothalamus, that are involved in the processing of emotional stimuli. Some brainstem areas, as the periaqueductal gray, on serotoninergic and noradrenergic nuclei (i.e., raphe complex and locus coeruleus), and several sensory and motor nuclei also express NOP receptors (for a review, see [5]). In contrast to the expression of NOP receptors, the distribution of the ppN/OFQ mRNA is almost limited to the limbic areas [6, 7]. 

In the periphery, NOP receptor has been detected in the peripheral nervous system and several organs, such as rat intestine and vans deferens [8], retina [9], heart [10], porcine gastrointestinal tract and kidney [11], and several guinea-pig ganglia. However, one of the principal locations of the NOP receptor in the periphery is the immune system. mRNA transcripts for the NOP receptor have been detected in mouse splenic lymphocytes (CD4^+^, CD8^+^, CD4^−^, and CD8^−^) [12]. In the human immune system, NOP receptors are also expressed in lymphocytic B and T-cell lines, monocytic cell lines, circulating lymphocytes, and monocytes [13, 14]. Taken together, this wide central and peripheral distribution of NOP expression may explain the broad spectrum of biological actions of this peptidergic system. In fact, *in vivo*, experimental studies have demonstrated that N/OFQ modulates a variety of biological functions, such as nociception [1, 2], food intake [15], learning and memory processes [16], spontaneous locomotor activity [2], rewarding actions of opioids [17] and ethanol [18], and responses to stress and anxiety [19]. Peripheral effects, such as hypotension, bradycardia [20], diuresis [21], inhibition of gastrointestinal [11] and airway [22] motility, and/or some reflexes such as coughing [23] and the micturition reflex [24] have also been reported for N/OFQ. Most recently, the effects of N/OFQ-NOP receptor system in modulating immune functions have received particular attention, in special its involvement in sepsis, inflammatory, and autoimmune diseases. In this review, we focus on summarizing the experimental evidence that suggests an important role played by this peptidergic system in the modulation of the immune functions, and we highlight the potential therapeutic effects of NOP ligands in the treatment of diseases which involve immune responses.

## 2. The Relationship between N/OFQ and Immune System

Neuropeptides have been described as important source of immunoregulatory molecules [25–27]. In this line, several evidence suggests that N/OFQ-NOP pathway may act as a potent regulatory during immune response. 

In 1995, Halford and colleagues [12] reported for the first time the expression of NOP receptors in mouse lymphocytes, while at the same year, Wick et al. [14] described the expression of NOP receptor mRNA in human peripheral blood lymphocytes and lymphocytic cell lines. In agreement, in 1998, Peluso and colleagues [13] detected the expression of mRNA NOP receptor in the human normal circulating lymphocytes and also in monocytes. Still in 1998, Pampusch et al. [28] demonstrated that NOP receptors are also expressed in the porcine immune cells and, in 2000, the same research group reported the expression of NOP receptor and ppN/OFQ mRNA in porcine lymphoid tissue, such as thymus, lymph nodes, spleen, and freshly isolated splenocytes [29]. Later, it was demonstrated that high-affinity binding sites for N/OFQ are distributed in human mononuclear [30] and polymorphonuclear leukocytes [31, 32]. Additionally, the incubation of human neutrophils with N/OFQ elicited intracellular signaling which was mimicked by the NOP synthetic agonist, Ro 64-6198, thus reinforcing that NOP receptors are present and functional in human neutrophils [32]. 

In 2002, for the first time, it was reported that the peripheral blood mononuclear cells transcribe ppN/OFQ [30]. Additionally, it was reported that N-formyl-methionine-leucine-phenylalanine-(fMLP-) stimulated polymorphonuclear neutrophils rapidly secrete N/OFQ by exocytosis, indicating that at least in neutrophils this neuropeptide is stored in preformed vesicles rather than *de novo* synthesized [32]. Nowadays, it is accepted that numerous immune cells were found to secrete N/OFQ, thus pointing to these inflammatory cells as a source of N/OFQ. Importantly, several inflammatory mediators including bacterial products (i.e., lipopolysaccharide, LPS) and cytokines could modulate both the transcription of ppN/OFQ mRNA and its protein expression [33, 34]. Therefore, N/OFQ seems to act as a paracrine, autocrine, and/or endocrine immunomodulator, since activated immune cells led to the induction of NOP receptor mRNA, and the same activated cells were able to express N/OFQ.

Based on *in vitro* and *in vivo* studies, many biological actions were reported for N/OFQ and NOP receptor system in modulating immune functions. *In vitro* studies have shown that N/OFQ evokes chemotaxis of polymorphonuclear [31] and human monocytes [35], and when injected in murine air pouches (an animal model to study leukocyte infiltration *in vivo*), N/OFQ elicits leukocyte infiltration in a concentration-dependent manner [31]. 

Further studies aiming to investigate the mechanisms by which N/OFQ stimulates chemotactic responses suggest that these effects are mediated by NOP receptors, since the selective blockade of NOP receptors prevent these actions [35]. It was found also that N/OFQ-stimulated polymorphonuclear neutrophils infiltration was inhibited by treating mice with a synthetic analog of the aspirin-triggered lipid mediator 15-epi-lipoxin A_4_ [31]. Taken together, these data suggest that N/OFQ, via activation of NOP receptor signaling, acts as a potent chemoattractant, possibly due to the involvement of arachidonic acid products. 

 Evidence from the literature also suggests that N/OFQ-NOP receptor system plays a role in regulating chemoattractant responses of leukocytes through chemokine suppression [36]. Chemokines are now known to be involved in mediating chemotaxis, integrin activation and leukocyte-endothelial cell interactions, leukocyte degranulation, and release of inflammatory mediators and angiogenesis ([37, 38]; for a review, see [39]). Kaminsky and Rogers [36] reported that N/OFQ suppresses the production of CCL2/MCP-1 and CCL5/RANTES chemokine protein in both primary CD14^+^ human monocytes and monocyte-like cell lines. However, this suppression does not seem to be at the level of transcription, as N/OFQ treatment did not alter the mRNA levels for these chemokines in monocytes.

A series of data support a modulatory effect of N/OFQ under cellular immunity, as summarized below. Peluso and colleagues [40] demonstrated that *in vitro* N/OFQ could decrease the proliferative response of human peripheral blood mononuclear cells stimulated with phytohemagglutinin. Miller and Fulford [34] have found that both the IL-2 production and T lymphocyte proliferation induced by the mitogen Con A were inhibited by N/OFQ. 

Elegant data described by researchers from McLeod's Laboratory have elucidated the mechanism by which N/OFQ modulates human T-cell response. First, the authors showed that N/OFQ inhibits the proliferative response of T cells activated by staphylococcal enterotoxin B (SEB) presented by Chinese Hamster Ovary (CHO) cells transfected with human HLA DR4 and CD80 [41], and in a subsequent work, they showed that the suppressive effect of N/OFQ on the T-cell proliferation could be blocked by the NOP receptor antagonist UFP-101, an anti-CD80 antibody, an inactivator of transforming growth factor-*β* (TGF-*β* LAP), L-NAME, and L-NMMA (both NOS inhibitors). They also have shown that N/OFQ induces the expression of the immunosuppressive modulator indoleamine 2,3-dioxygenase (IDO) [42]. In view of the factors modulating IDO expression, it was suggested that N/OFQ decreases T-cell proliferation through IDO expression by a mechanism involving IFN-*γ*, TGF-*β*, prostaglandin and nitric oxide.

The ability of N/OFQ-NOP receptor system in modulating humoral immunity was also described in the literature. Recently, it has been demonstrated that N/OFQ, added *in vitro* to murine spleen cells at picomolar concentrations, caused a significant suppression of antibody formation against sheep red blood cells, which was reversed by NOP antagonists [43]. In addition, N/OFQ given *in vivo* by osmotic pump for 48 h suppressed the capacity of spleen cells cultured *ex vivo* to produce anti-sheep red blood cell antibody [43]. These studies showed that N/OFQ directly inhibits an adaptive immune response, that is, antibody production, both *in vitro* and *in vivo*.

In agreement with the evidence for an immunosuppressive effect evoked by N/OFQ, Du and colleagues [44] have shown that in normal rats the intracerebroventricular administration of N/OFQ, at nmol doses, reduced the NK cell activity. However, in rats under surgical trauma, N/OFQ antagonised the immune function depression, and this anti-immunosuppression evoked by N/OFQ was completely reversed by the pretreatment with the antisense oligonucleotide to NOP receptor mRNA [44]. Taken together, a growing body of evidence suggests a complex role for N/OFQ in modulating, by inhibiting or activating, immune functions depending on the nature of the stimuli.

## 3. The Involvement of Inflammatory Mediators in N/OFQ-Evoked Immune Actions

It is well established that regulation of immune and inflammatory responses depends on cytokines signaling. Therefore, it is expected that N/OFQ evokes immune responses as a result of the modulation of cytokine activity or expression. However, it is possible that the altered expression of cytokines is due to the NOP receptor signaling-induced production of regulatory mediators which, in turn, lead to altered levels of cytokines (for a review, see [39]). 

There is no consensus regarding the role of N/OFQ on inflammatory and immune responses. In fact, Goldfarb and colleagues [45] demonstrated that in C57BL/6J mice the intraperitoneal injection of N/OFQ (55 nmol/kg) 30 min before challenge with Staphylococcal enterotoxin A (SEA, 5 *μ*g) caused a significant increase in levels of spleen TNF-*α* and IFN-*γ* mRNA, without causing any significant effects on IL-1*β* and IL-2 mRNA levels. Moreover, mice lacking the N/OFQ precursor gene showed diminished TNF-*α* and IFN-*γ* transcripts in the spleen in response to SEA challenge, suggesting that N/OFQ system can exacerbate the immune function. 

By contrast, following the induction of trauma in rats, Zhao and colleagues [46] found typical robust increases in both TNF-*α* and IL-1*β* transcripts in peritoneal macrophages, but when N/OFQ was intracerebroventricularly administered, both TNF-*α* and IL-1*β* expression was attenuated in these cells. Additionally, N/OFQ immunoreactive cells and mRNA for NOP receptor were significantly reduced after trauma in cerebral cortex, hippocampus, and hypothalamus of rats [46]. Still under traumatic stress, high levels of IL-1*β* transcript in hippocampus were antagonised by icv administration of N/OFQ, and these effects were reversed by the treatment with an NOP receptor antagonist [47], thus suggesting that the effects of N/OFQ in the cytokines expression could depend on the tissues, pretreatment time, dose and via of N/OFQ administration, and nature of stimuli [39]. Obviously, further studies at this point should be performed in order to give additional information about the role played by the N/OFQ-NOP receptor system in the modulating cytokines expression. 

It is worthy of mentioning that the inflammatory process is a necessary step for induction of protective immunity and to contain the infections, but can also lead to dangerous effects, if uncontrolled. Hence, an imbalance between proinflammatory and anti-inflammatory mediators, between Th1/Th17 effector cells and T regulatory cells may result in hyperinflammatory or autoimmune diseases. Considering the bidirectional relationship between the N/OFQ-NOP and the immune system, it is possible that NOP receptor signaling modulates the outcome of some inflammatory diseases including sepsis, autoimmunity, and possibly the transplantation rejection, a condition not investigated yet.

## 4. N/OFQ-NOP Receptor System and Inflammatory and Autoimmune Diseases

The complex syndrome of sepsis is characterized by impaired recruitment of neutrophils, inability of the immune system to limit bacterial spread during an ongoing infection and systemic inflammation associated with deleterious accumulation of neutrophils in vital organs, especially the lung that culminates in multiple organ failure [48–50]. The systemic inflammatory response syndrome (SIRS), hallmark sign of sepsis, is characterized by a systemic inflammatory response with massive release of proinflammatory cytokines such as TNF-*α*, IL-1*β*, chemokines, nitric oxide, leukotrienes, reactive oxygen species [51, 52]. This response is counterregulatory by a compensatory anti-inflammatory response syndrome (CARS) through the secretion of inhibitory molecules such as IL-10 and TGF-*β*. It has been suggested that in septic patients a delicate balance between the pro and anti-inflammatory mediators are decisive to the outcome of disease [53], since the proinflammatory phase is necessary for both the recruitment of neutrophils and induction of microbicidal activity but also responsible for the failure of neutrophils migration to the infectious focus. In addition, it may be related to subsequent immunosuppression [49, 54]. 

Using the caecal ligation and puncture (CLP) model of sepsis, our group have demonstrated that the administration of the NOP receptor antagonist UFP-101, immediately after surgery, promoted a significantly reduction on mortality rate of septic rats compared with CLP-untreated rats [55]. In this study, we observed that the protective dose of NOP antagonist was able to prevent the massive influx of inflammatory cells into both the lung and bacterial dissemination. These protective effects were associated with significant decrease in plasmatic levels of TNF-*α*, IL-1*β*, and CCL2 [55]. It should be mentioned that this beneficial effect of UFP-101 was evident after a single drug injection after induction of CLP, suggesting that the N/OFQ-NOP system plays an important role in modulating the early phases of the septic process. The molecular mechanisms by which the NOP receptor antagonist, UFP-101 have protected septic rats are under investigation by our group. In our study, we did not evaluate the role of nitric oxide and other regulatory molecules such as programmed death-1 (PD-1)/PD-1 ligands, TGF-*β*, and CLTA-4 (Cytotoxic T-lymphocyte antigen 4). Although we have detected a decreased number of bacteria in the blood, it was not investigated whether NOP antagonist modulates directly or indirectly the bactericidal activity of neutrophils. 

Additional data have supported the idea that high levels of N/OFQ in septic patients may contribute to its pathogenesis. Despite the small size of the sample, it was found that septic patients who died within 30 days (*n* = 4) displayed higher N/OFQ plasmatic levels compared with survivors (*n* = 17) [56]. However, when evaluating the role of N/OFQ-NOP receptor system in a complex event such as sepsis, we should be aware of the broad range of effects evoked by NOP receptor signaling, that contribute to the septic condition, such as impairment of cardiovascular performance and tissue perfusion, beside the immune responses herein discussed (for a review see [57]). Very recently, Stamer and colleagues [58] have shown that peripheral blood cells from patients with sepsis had higher N/OFQ and lower ppN/OFQ mRNA expression than healthy controls. Again, these observations suggest the involvement of the NOP-N/OFQ system in inflammation and impaired immune function in sepsis.

Corroborative data showing the involvement of N/OFQ-NOP system in the physiopathology of other immune disturbances have been described. In this regard, Fiset and colleagues [32] found high levels of N/OFQ in the synovial fluid but not in the plasma of patients with arthritis. In view of the presence of N/OFQ and polymorphonuclear cells accumulation in the inflammatory synovial cavity of arthritic patients, further studies are imperative to investigate the involvement of N/OFQ-NOP receptor at the beginning of the inflammatory process.

Using a mouse model of bowel disease induced by the injection of dextran sulfate sodium, Kato and colleagues [59] demonstrated that both the inflammatory response and severity of disease were associated with the upregulation of N/OFQ expression. Furthermore, NOP knockout mice did not develop colitis, thus indicating the participation of N/OFQ in disease [59]. Regarding the histopathological findings, the authors found that the number of T lymphocytes (T CD4^+^ and T CD8^+^), B lymphocytes, macrophages, and neutrophils present in the colonic mucosa of dextran sulphate sodium-treated NOP-deficient mice was significantly lower than the number of those found in wild-type animals. 

Concerning Parkinson's disease, the second most common age-associated progressive neurodegenerative disorder, the inflammation, oxidative stress, and microglia-mediated neurotoxicity of dopaminergic neurons in the substantia nigra are considered the hallmark of disease [60–63]. Some findings for neuroinflammatory mechanisms leading to the immunopathology of Parkinson's disease include microgial activation, secretion of TNF-*α*, IL-1*β*, IL-2, IL-6, and CCL5, antibody against different components of dopaminergic neurons, expression of inducible nitric oxide synthase, astrogliosis, shift to Th1 phenotype, increased number of activated T cells, and other inflammatory markers [60–62, 64–69]. Several preclinical studies reported by Professor's Morari's group have suggested the participation of N/OFQ in the physiopathology of Parkinson's disease [70–74]. Interestingly, an enhancement of N/OFQ expression and release in 6-hidroxydopamine hemi-lesioned rats was detected in the lesioned substantia nigra compared with the unlesioned, indicating that parkinsonism may be associated with overactivation of NOP receptor signaling [71]. Furthermore, the pharmacological blockade of NOP receptor signaling attenuates parkinsonian-like behavior in 6-hydroxydopamine hemi-lesioned, haloperidol, and reserpine-treated rodents, whereas deletion of the NOP receptor gene conferred mice protection from these symptoms [71, 74, 75]. Corroborating to the view that N/OFQ-NOP receptor system plays an important role in Parkinson's disease, very recently, Marti and colleagues [76] found increased levels of N/OFQ in the cerebrospinal fluid of parkinsonian patients compared with nonparkinsonian.

## 5. Mechanisms Underlying N/OFQ-Induced Immune Regulation

The OFQ/N system modulates many functions in a variety of immune cells including monocytes, macrophages, neutrophils, mast cells, and lymphocytes by a mechanism not clearly elucidated yet.  Table 1 summarizes some of these functions, the participation of N/OFQ in the neuroimmune axis, and the mechanisms underlying some immunoregulatory activities triggered by NOP receptor activation. 

NOP receptor together with the classical opioids (*μ*, *κ*, *δ*) receptors belongs to the G-protein-coupled receptor (GPCR) family, which plays a vital role in the transduction of signals regulating several effectors. Regarding to N/OFQ, some authors have described that the NOP activation by its natural ligand or agonists induces the activation of K^+^ conductance and inhibition of voltage-gated Ca^2+^ channels and either augments (via activated *α*-subunit of Gs class of G-protein) or decreases (via the activated form of the G_i_/G_o_) cAMP formation in a variety of cells including immune cells [1, 2, 4, 86–88]. N/OFQ also induces stimulation of phospholipase C (via *α*-subunit of G_q_), which leads to 1,4,5-triphosphate (IP_3_) and diacylglycerol production and also to Ca^2+^-dependent protein kinase C (PKC) activation (for a review, see [83, 89, 90]). It was also demonstrated that in CHO cells the type of G-protein involved in PKC activation by N/OFQ were a G_i/o_ G-protein [91]. Moreover, N/OFQ modulates extracellular signal-regulated kinase (ERK), p38, c-Jun N-terminal Kinase (JNK) isoforms of mitogen-activated protein kinase (MAPK) [82, 90, 92, 93], and the transcription of a variety of genes involved in immune and inflammatory responses [83, 94]. It had been suggested that the signal transducer and activator of transcription (STAT3) may be involved in the transduction of NOP signaling [90, 95]. 

Considering the glia-immune cells communication, it was demonstrated that LPS, IL-1*β*, and TNF-*α* increase the levels of N/OFQ mRNA and immunoreactivity in rat astrocytes in culture by a mechanism dependent of the activation of ERK 1/2, p38 MAP kinases and the transcription factor CREB. It was demonstrated that NF*κ*B pathway appears to be involved in the induction of N/OFQ transcription by LPS [33]. N/OFQ has been shown to cause I*κ*B kinase (IKK) phosphorylation and I*κ*B degradation in SH-SY5Y human neuroblastoma cells [84]. Recently, Donica and colleagues [85] showed that N/OFQ increases the nuclear translocation, binding to DNA, and activation of transcription. Hence, the activation of NF*κ*B by N/OFQ may be critical for many immune functions.

## 6. Relationship between N/OFQ and Classical Opioids in the Regulation of Immune Functions

As commented above, N/OFQ belongs to the opioid family, but despite the structural homology between ligands and receptors, there is no evidence that N/OFQ activates opioid receptors, neither classical opioid ligands activate NOP receptor [83]. Importantly, NOP and ppN/OFQ mRNA are found expressed constitutively in distinct cells of the immune system [1, 45, 96], while *kappa* opioid receptors are constitutively expressed in lymphocytic cells [97], but this is not the case of mu and delta opioid receptors [13, 98]. Additionally, beta-endorphin and dynorphin are expressed and secreted by immune cells, including T cells, and upregulated in these cells by various stimuli [99–103]. It is wellknown that cytokines, mediators typically released from immune cells, are potent regulators of mu-opioid receptor gene expression. In fact, mu receptors are not constitutively expressed in immune cells, but these receptors are upregulated in T cells and B cells following pretreatment with interleukin 4 (IL-4) and TNF-*α* [104, 105]. Thus, suggesting a putative role of classical opioids in the regulation of the immune functions. 

It is well documented that opioid peptides cause immunosuppressive effects in some assays and stimulatory actions in others [106–108], however, morphine, which is a preferred mu receptor agonist, generally has been found to be immunosuppressive in a variety of assays [109]. 

The current review has showed that N/OFQ has immunomodulatory actions, by stimulating or inhibiting the immune system. However, little information is still available in the literature regarding the relationship between N/OFQ and classical opioid systems. Anton and colleagues [43] showed that N/OFQ inhibits antibody formation in a naloxone-insensitive manner, thus suggesting that N/OFQ effects, at least in this particular case, are not mediated by classical opioid receptors [43]. Another recent evidence that show no interaction between N/OFQ-NOP receptor system and the classic opioid system is coming from a clinical study, in which patients suffering from cancer who were taking opioid medication (morphine or equivalents) did not display any significant alterations in NOP and ppN/OFQ expression compared with those who did not receive opioids pretreatment [58]. These observations suggest that, despite the immunosuppressive effects of opioid in addicted patients [110], the mechanisms by which classical opioids affect immune functions, especially in the periphery, did not seem to be related to the N/OFQ-NOP receptor system. 

Considering the expression of classical opioids (ligands and receptors) and N/OFQ-NOP receptor in the central nervous system, beside the immune cells, a putative neuroimmune cross-talk could be suggested. It is accepted that classical opioids depress the immune function, due to the activation of the hypothalamic-pituitary-adrenal axis (HPA), thus increasing corticoid hormone production [111]. Some studies have suggested that N/OFQ activates the HPA axis. In fact, intracerebroventricular injection of N/OFQ in rats led to increased plasma adrenocorticotropic hormone (ACTH) concentrations [112] and corticosterone [113] under resting conditions. N/OFQ administered to rats under mild stress enhanced the raised ACTH response to stress and prolonged the higher concentrations of corticosterone, while UFP-101, an NOP receptor antagonist, significantly attenuated plasma ACTH and corticosterone compared to saline injection in LPS-treated rats [114]. However, under a more stressful stimulus, such as restraint, central injection of N/OFQ did not affect the elevated plasma concentrations of ACTH or corticosterone [112]. 

Additionally, Leggett et al. [114] found increased corticotrophin-releasing factor (CRF) and corticotrophic pro-opiomelanocortin (POMC; a protein precursor of ACTH, beta-endorphin, and Met-enkephalin) mRNA into the parvocellular paraventricular nucleus expression due to LPS challenge compared to non-LPS treated rats. Additionally, the intracerebroventricular administration of UFP-101 in LPS-treated rats was associated with increased POMC mRNA expression 4 h after injection and a clear trend towards increased parvocellular CRF mRNA. Taken together, a neuroimmune cross-talk between the N/OFQ-NOP receptor and classical opioid systems seems to be occurring and could be involved in the modulation of the immune responses.

## 7. Concluding Remark

In conclusion, there is no consensus regarding the role of N/OFQ-NOP receptor system in modulating inflammatory and immune responses. In fact, some studies have shown that N/OFQ is able to evoke chemotaxis, inhibit cellular and humoral immunity, and increase or suppress the expression of proinflammatory cytokines, such as TNF-*α* and IFN-*γ*. Certainly, these immunological effects evoked by this peptidergic system make the NOP receptor an attractive therapeutic target for the treatment of inflammatory diseases, such as sepsis, rheumatoid arthritis, colitis, and other immunological disturbances. Interesting enough, preclinical findings from animal models of sepsis, colitis, and Parkinson pointed to the blockade of NOP receptor signaling as beneficial to diseases' outcome. Additional support to the involvement of N/OFQ in mediating these pathologies was found in human body fluids from patients suffering from sepsis, arthritis, and Parkinson, which displays increased N/OFQ levels. Thus, showing the vast range of immune functions evoked by N/OFQ, an extensive research in basic and clinical sciences is mandatory to further investigate the potential therapeutic effects of NOP ligands in the treatment of inflammatory diseases.

## Figures and Tables

**Table 1:** Mechanisms underlying some immunoregulatory activities triggered by NOP receptor activation.

Biological effects related to N/OFQ or its interaction in the neuroimmune axis	Mechanisms	Animal conditions or cell type	Ref
Bacterial products (LPS) and inflammatory cytokines (IL-1*β* and TNF-*α*) increase the expression of N/OFQ in astrocytes	Dependent on ERK, p38 MAPK, and NF-*κ*B activation	Primary rat astrocytes culture	[33]

LPS induce N/OFQ expression in dorsal root ganglion (DRG) neurons	The complex TLR-4-MD-1 seems to be a functional receptor of neurons for LPS	DRG neurons obtained from mice	[77]

N/OFQ induces vasodilatation and hyperemia in acutely inflamed rat knees	Dependent of mast cells, and circulating leukocytes, which produces proinflammatory factors that stimulate capsaicin-sensitive nerves leading to release of SP, CGRP and VIP	Anesthetized rats (kaolin/carragee an arthritis model)	[78]

Increased vascular permeability in rat skin by local application of nociceptin	Histamine released from mast cells	Wistar rats, peritoneal mast cells isolated	[79]

N/OFQ i.v. injection induces hypotension, vasodilatation, vascular permeability, leukocytes rolling, and adhesion	Vasodilation and inflammation dependent, of histamine released by mast cells	Wistar rats	[80]

N/OFQ elicits in mice itch and leukotriene B_4_ production	Not clear	ICR mice, keratinocytes from mice	[81]

N/OFQ induces chemotaxis of neutrophils *in vitro* and leukocytes recruitment *in vivo *	The chemotaxis were Ca^2+^ independent, and leukocytes infiltration into air pouches cavities were inhibited by 15-Epi-Lipoxin A_4_ treatment	Human PMN-isolated and murine air pouches model	[31]

Human neutrophils secrete preformed N/OFQ upon degranulation induced by microbial-derived *N*-formylated protein	N/OFQ prevents cAMP elevation in fMLP-stimulated PMNs acting as an activating signal	Human neutrophils	[32]

NOP-deficient mice developed attenuated colitis when orally treated with dextran sulfate sodium	Decreased expression of MadCAM-1 and significant reduction in the number of lymphocytes, macrophages and neutrophils in colonic mucosa	NOP deficient and wild-type C57BL6 mice	[59]

Pharmacological blockade of NOP receptor decreases rate mortality and systemic inflammation in septic rats	Control of bacteria spread (peritoneal cavity and blood) and decreased lung infiltration and serum levels of TNF-*α*, IL-1*β*, and CCL2	Rats subjected to CLP	[55]

N/OFQ decreases SEB-activated T-cell proliferation	Induction of active IDO expression in T cells by a mechanism involving IFN-*γ*, TGF-*β*, prostaglandin, and nitric oxide	SEB-activated CD4^+^ T cells	[42]

Modulation of genes transcription involved in the neuroimmune axis functions	N/OFQ induces cell signaling via several intracellular pathways leading to MAP kinase activation, PKC activation, NF*κ*B nuclear translocation, and hence gene transcriptions	Human neuroblastoma SH-SY5Y cells, CHO transfected cells	[82–85]

CCL2: chemokine (C-C motif) ligand 2; CGRP: calcitonin gene-related peptide; CLP: cecal ligation and puncture; ERK: extracellular signal-regulated kinase; fMLP: formyl-methionyl-leucyl-phenylalanin; IDO: indoleamine 2,3-dioxygenase; MAdCAM-1: mucosal addresin cell adhesion molecule-1; MAPK: mitogen-activated protein kinase; MD-1: myeloid differentiation protein-1; NF*κ*B: nuclear factor Kappa B; PMN: polymorphonuclear neutrophil; SP: substance P; TLR4: toll-like receptor 4; VIP: vasoactive intestinal peptide.
